# Long-term prognostic value of computed tomography-based attenuation correction on thallium-201 myocardial perfusion imaging: A cohort study

**DOI:** 10.1371/journal.pone.0258983

**Published:** 2021-10-26

**Authors:** Jei-Yie Huang, Ruoh-Fang Yen, Chun-Kai Huang, Chia-Ju Liu, Mei-Fang Cheng, Kuo-Liong Chien, Yen-Wen Wu

**Affiliations:** 1 Department of Nuclear Medicine, National Taiwan University Hospital and National Taiwan University College of Medicine, Taipei, Taiwan; 2 Institute of Epidemiology and Preventive Medicine, College of Public Health, National Taiwan University, Taipei, Taiwan; 3 Department of Internal Medicine, National Taiwan University Hospital and National Taiwan University College of Medicine, Taipei, Taiwan; 4 Department of Nuclear Medicine, National Taiwan University Hospital, Yun-Lin Branch, Yun-Lin County, Taiwan; 5 Department of Nuclear Medicine, Far Eastern Memorial Hospital, New Taipei City, Taiwan; 6 Division of Cardiology, Cardiovascular Medical Center, Far Eastern Memorial Hospital, New Taipei City, Taiwan; 7 National Yang Ming Chao Tung University School of Medicine, Taipei, Taiwan; Rutgers New Jersey Medical School, UNITED STATES

## Abstract

**Background:**

Myocardial perfusion imaging (MPI) is a well-established diagnostic tool to evaluate coronary artery disease (CAD) and also an effective prognostic tool for patients with CAD. However, few studies investigated the prognostic value of attenuation correction (AC) in MPI, and the results were controversial.

**Objectives:**

To investigate the prognostic value of computed tomography (CT)-based AC thallium-201 (Tl-201) MPI.

**Methods:**

A total of 108 consecutive patients who underwent Tl-201 MPI and received coronary angiography within 90 days were included. Medical records were reviewed and missing information was completed after telephone contact. The prognostic value was evaluated by Kaplan-Meier analysis, univariable and multivariable Cox proportional hazards model.

**Results:**

After a mean follow-up of 7.72 ± 3.72 years, 27 patients had died, 41 had been readmitted for cardiovascular (CV)-related events and 44 had reached the composite of death plus CV-related re-admission. Kaplan-Meier curves for all-cause mortality for SSS with a cutoff value of 13 for AC and 16 for non-AC (NAC) images showed a significant difference between the two curves for both AC and NAC images (p = 0.011 for AC and p = 0.021 for NAC). In the multivariable model, SSS and SRS showed similar independent predictive values in predicting all-cause mortality and composite of all-cause mortality plus CV-related re-admission, in both AC and NAC images. Subgroup analysis implicated that AC MPI possibly provided better risk stratification in obese patients.

**Conclusion:**

CT-based AC and NAC MPI showed similar value and were the only significant predictors for the composite of mortality and CV events.

## Introduction

Myocardial perfusion imaging (MPI) is a well-established diagnostic tool to evaluate coronary artery disease (CAD) [1]. However, the issue of attenuation artifacts is still important, especially when using low-energy radiotracer [2–6]. Attenuation correction (AC) corrects this artifact and improves diagnostic performance of MPI [5, 7, 8]. MPI has also been shown to be an effective prognostic tool for patients with CAD [9, 10], however few studies have investigated the prognostic value of attenuation correction (AC) [11–17]. Some studies have reported that AC can result in more efficient risk stratification compared with non-AC (NAC) by demonstrating greater event rates in AC images compared with the same scoring group of NAC images, and significant hazard ratios (HR) of AC images [11, 12, 14]. Other studies have shown similar event rates between the same AC and NAC scoring groups and significant HR of NAC images [13, 15].

The first computed tomography (CT)-base AC study was conducted in 2005 with combined single photon emission computed tomography/computed tomography (SPECT/CT) [18]. CT-based AC MPI provided a high-resolution transmission map in a short time [19]. With regards to the prognostic effect of CT-based AC, two of three studies have demonstrated similar value between CT-based AC and NAC images [13, 15].

The aim of this study was to investigate the prognostic value of CT-based AC and NAC thallium-201 (Tl-201) MPI. The primary endpoint was all-cause mortality. The secondary endpoints were cardiovascular (CV)-related re-admission, heart failure (HF)-related admission, and a composite of all-cause mortality and CV-related re-admission.

## Methods

### Study design and study population

This study was approved by the Research Ethics Committee of National Taiwan University Hospital and complied with the Declaration of Helsinki (approval number: 201106041RC and 201701112RINA). The need for written informed consent was waived due to the retrospective nature of the study. Patients received sequential coronary angiography (CAG) due to abnormal or uncertain MPI results (≤90 days) were retrospectively enrolled and the prognostic performance of Tl-201 MPI with or without AC was evaluated.

A total of 2,603 consecutive patients were referred for MPI at the Division of Nuclear Medicine, National Taiwan University Hospital Yun-Lin Branch from January 2008 to June 2009. The inclusion criteria were patients (1) with known or suspected CAD, (2) who underwent CT-based AC MPI, and (3) who received coronary angiography (CAG) within 90 days after MPI. The exclusion criteria were (1) missing CAG reports, (2) patients who did not undergo CT-based AC for technical reasons, and (3) duplicate patients.

Clinical data including age, sex, height, weight, subjective symptoms, medical history, pharmacologic treatment, and conventional CV risk factors were surveyed at the time of the index MPI study. Baseline heart rate and blood pressure during MPI were also recorded. CV risk was assessed as the number of risk factors and calculated into Framingham general CV disease risk prediction score (FRS) [20]. A total of 108 subjects who underwent CAG within 90 days were enrolled for subsequent analysis.

### Image acquisition and reconstruction

Standardized stress Tl-201 MPI were performed based on the American Society of Nuclear Cardiology and the American Heart Association guidelines [21]. MPI was acquired at 5 minutes and again at 3 to 4 hours after an injection of 2.5–3 mCi Tl-201. Reinjections were performed if a severe perfusion defect was noted in the post-stress images. Electrocardiography (ECG)-gated SPECT (eight frames per cardiac cycle) was acquired on a dual-head SPECT/CT scanner (Symbia T2, Siemens Medical Solution Inc., Hoffman Estates, Illinois, USA) equipped with low-energy general purpose collimators using a noncircular 180° acquisition for 64 projections at 22 (at stress) and 30 (at redistribution) seconds per projection. Two energy windows were set at 72 keV (±15%) and 167 keV (±10%) of Tl-201. Emission images were processed using Syngo imaging software (Siemens) and stored in a 64 x 64 matrix [5].

A single low-dose breath-hold CT scan was performed after post-stress emission acquisition. In addition, a CT attenuation map was reconstructed using an average CT. Details of the acquisition protocols and parameters have been described previously [5]. CT images were reconstructed onto a 512 x 512 matrix. Attenuation maps were generated at both low- and high-energy spectrums, and attenuation coefficients were generated pixel by pixel. The emission and transmission images were manually matched to avoid mis-registration artifacts [22, 23]. Scatter correction was applied using a triple energy window method [24]. Both NAC and AC emission images were reconstructed using ordered-subsets expectation maximization reconstruction method with 3D collimator beam modeling (Flash-3D) with a Gaussian filter (eight iterations, eight subsets; full-width half-maximum 8.4 mm), and by resampling the data along the short, vertical long, and horizontal long axes for display [25, 26]. The estimated effective radiation dose for the whole study was 16.0–19.5 millisievert.

### Imaging analysis

Two experienced nuclear medicine physicians interpreted the MPI images with consensus. The images were presented in a random sequence for the physicians to interpret, and the physicians were blinded to the patient’s history, CAG findings, and information from CT. The myocardium was divided into standardized American Heart Association 17 segments, and each segment was scored in a semi-quantitative manner using a standard 5-point scoring system [27]. We graded NAC images segment by segment first then a combination of AC and NAC images together. The summed stress score (SSS), summed rest score (SRS), and summed difference score (SDS) were obtained. Non-perfusion abnormalities (transient left ventricle dilation, lung/heart ratio) and gated information (volume, ejection fraction and wall motion) were not taken into account during perfusion scoring due to the assumption that these data would not be affected by AC [28].

We calculated receiver operating characteristic (ROC) curve and Youden index for the optimal cutoff value for AC and NAC images. An SSS of 13 was chosen as the optimal cutoff for AC and an SSS of 16 was chosen for NAC images. An SRS of 6 was chosen as the optimal cutoff for AC and NAC images. We also analyzed the prognostic value of MPI with traditional cutoff values for the severity of MPI, with a score < 4 considered to be normal; 4 to 8 as mildly abnormal; 9 to 13 as moderately abnormal; and > 13 as severely abnormal, respectively for SSS and SRS [29].

### Clinical follow-up

All patients received CAG, and CAD was defined as > 50% stenosis of the left main artery (LM) and 70% stenosis of the left anterior descending coronary artery (LAD), left circumflex coronary artery (LCx) or right coronary artery (RCA). Medical records were reviewed until December 2018. We recorded the causes of mortality if available, major adverse cardiac events (MACEs; cardiac death and nonfatal myocardial infarction), CV-related re-admission and HF admission. The definition of CV-related re-admission was the first time the patient was admitted due to CV reasons, with discharge principal diagnosis of CAD, myocardial infarction, heart failure and arrhythmia, after the time he or she was admitted for index coronary angiography. If data on events and clinical follow-up information before December 31, 2018 were missing, the patients were followed up by telephone with scripted interviews. Details including death or survival, cause of death, time of death, whether they received regular medical follow-up and follow-up location were recorded. Data were censored at the first cardiac event or at the time of last follow-up.

The primary study endpoint was all-cause mortality. The secondary endpoints were CV-related re-admission, HF admission and the composite of death and CV-related readmission.

### Statistical analysis

Descriptive analysis was performed according to outcome (all-cause mortality) status. The Student’s t-test and Fisher’s exact test were used to compare differences between mortality and non-mortality. Semi-quantitative data were expressed as mean ± standard deviation. Comparisons between two AC and NAC were performed using the non-parametric paired samples *t*-test.

Kaplan-Meier curves according to the cutoff values were drawn, and the log-rank test and Wilcoxon test were used for comparisons between them when appropriate [30]. A Cox proportional hazards model was used to investigate the prognostic value of each clinical and scintigraphic variable (univariable analysis). Statistically significant variables in univariable analysis were then entered into the multivariable Cox regression model [31, 32]. Due to the significant correlation between SSS, SRS, SDS evaluated by AC and NAC MPI, we analyzed AC SSS, NAC SSS, AC SRS, NAC SRS, AC SDS and NAC SDS in separate multivariable Cox regression models. Subgroup analyses according to body habitus (BMI cutoff value of < 27 and ≥ 27 kg/m^2^) and discordant or concordant findings of AC and NAC images were done to find out which subgroup would benefit most from AC.

A two-sided P-value < 0.05 was considered to be statistically significant. Statistical analysis was performed with commercial software (SAS version 9.4, SAS Institute Inc., North Carolina, USA).

## Results

### Patient

A total of 108 patients [36 women (33.3%) and 72 men; mean age: 64.65 ± 10.53 years] who received CAG within 90 days were included in this study. No adverse events from MPI or CAG were noted. The detailed clinical characteristics of the patients were summarized in Table 1. Histories of CAD and coronary artery bypass graft surgery were recorded during history taking before MPI. Significant differences between the patients who died and survived were noted in age, BMI, left ventricular ejection fraction (LVEF), both AC and NAC SSS and diabetes. The MPI characteristics were summarized in Table 2. Significant differences were noted between AC and NAC images in all SSS, SRS and SDS.

**Table 1:** Detailed clinical characteristics of the patients by outcome status.

	Total	Death (+) n = 27	Death (-) n = 81	*p value*
	**mean (SD)**			
Age (years)	64.65 (10.53)	69.30 (8.41)	63.10 (10.76)	0.008
Body mass index (kg/m^2^)	25.90 (4.18)	23.97 (3.13)	26.55 (4.30)	0.005
LVEF (%)	59.81 (12.77)	52.68 (11.22)	62.10 (12.45)	0.001
	**Median (IQR)**			
AC SSS	11 (15)	17 (15)	9 (13)	0.005
NAC SSS	16 (15)	22 (10)	14 (13)	0.002
AC SRS	5 (10)	11 (9)	4 (7)	0.001
NAC SRS	8 (10)	13 (11)	5 (9)	<0.001
AC SDS	5 (7)	7 (7)	5 (6)	0.29
NAC SDS	6 (6.5)	7 (8)	6 (6)	0.58
	**n (%)**			
Women	36 (33.3%)	12 (44.4%)	24 (29.6%)	0.16
Clinical history				
Hypertension	81 (75.0%)	22 (81.5%)	59 (72.8%)	0.37
Diabetes	47 (43.5%)	18 (66.7%)	29 (35.8%)	0.005
Dyslipidemia	45 (41.7%)	13 (48.2%)	32 (39.5%)	0.43
Smoking	14 (13.0%)	3 (11.1%)	11 (13.6%)	0.74
Known CAD	49 (45.4%)	16 (59.3%)	33 (40.7%)	0.09
History of CABG	8 (7.4%)	3 (11.1%)	5 (6.2%)	0.41
PCI after MPI	52 (48.2%)	18 (66.7%)	34 (42.0%)	0.026
CABG after MPI	3 (2.8%)	1 (3.7%)	2 (2.5%)	1.00

AC: attenuation correction; CABG: coronary artery bypass graft; CAD: coronary artery disease; NAC: non-attenuation correction; PCI: percutaneous coronary intervention; SDS: summed difference score; SSS: summed stress score.

**Table 2:** MPI characteristics, including SSS and SDS in AC and NAC MPI.

	AC	NAC	AC	NAC	p value
	Mean(SD)		Median (IQR)		
SSS	13.57 (9.51)	16.55 (9.58)	11 (15)	16 (15)	*<0*.*001*
SRS	7.62 (7.38)	9.43 (8.17)	5 (10)	8 (10)	*<0*.*001*
SDS	5.95 (5.06)	7.12 (5.30)	5 (7)	6 (6.5)	*<0*.*001*

AC: attenuation correction; IQR: interquartile range; NAC: non-attenuation correction; SD: standard deviation; SDS: summed difference score; SSS: summed stress score.

Sixty-one patients (56.5%) were diagnosed with CAD by CAG, with stenosis affecting the LAD in 46 (42.6%), LCx in 33 (30.6%), and RCA in 37 (34.9%) patients. Of the patients with CAD, 23 (37.7%) had one-vessel disease, 21 (34.4%) had two-vessel disease, and 17 (27.9%) had three-vessel disease. Only 106 reference RCA angiographic results were analyzed due to technical failure of RCA angiography in two patients. Percutaneous coronary intervention, such as balloon angioplasty or stenting, surgical intervention, such as coronary artery bypass graft or medical treatment were decided under a mutual agreement between the patients and cardiologists. Fifty-two (48.1%) patients received percutaneous coronary intervention and 3 (2.8%) patients received bypass surgery after MPI. The overall diagnostic performance of MPI comparing AC with NAC was reported in previous study [5].

After a mean follow-up period of 7.72 ± 3.72 years, 27 (25%) patients had died, 41 (38%) had been readmitted for CV-related events (after the first CAG), 44 (41%) had reached the composite of death plus CV-related re-admission, and five (5%) had been admitted for HF.

### Survival analysis

Trends of higher mortality rates were noted in the more severe SSS and SRS groups in both NAC and AC images. However, no trends of increased or decreased mortality rates were noted in SDS 0–1, 2–4, 5–8, >8 groups in either NAC or AC images. The mortality rates in SSS, SRS 0–3, 4–8,9–13, >13 and SDS 0–1, 2–4, 5–8, >8 groups of AC and NAC images were shown in S1 Table.

**Fig 1:**
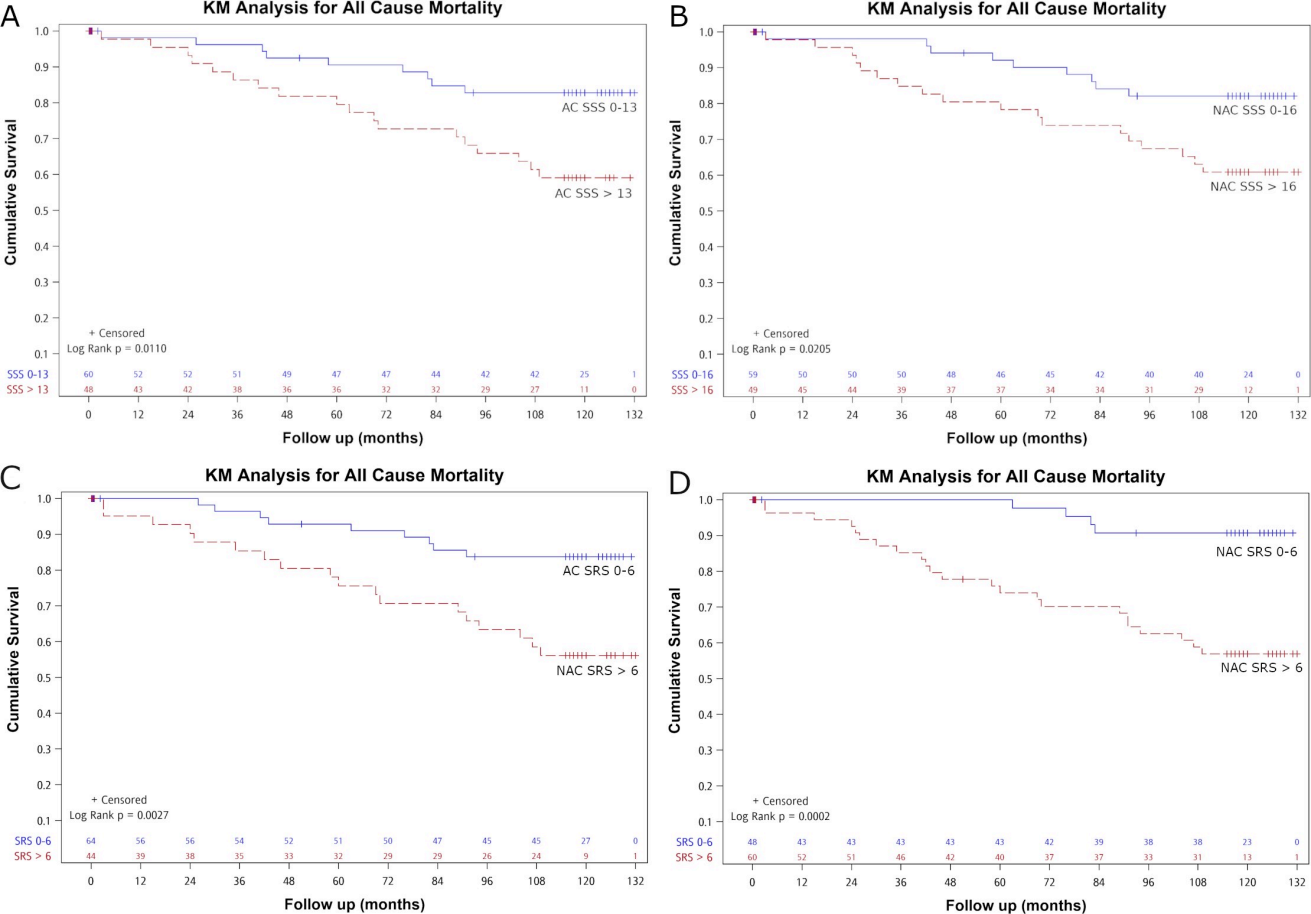
Kaplan-Meier (KM) analysis regarding all-cause mortality with cutoff values of SSS ≤ 13 versus SSS > 13 in AC (A), SSS ≤ 16 versus SSS > 16 in NAC (B), SRS ≤ 6 and SRS > 6 in AC (C) and NAC (D) images. Significant differences between survivor functions were noted between AC SSS ≤ 13 versus AC SSS > 13 (p = 0.011), NAC SSS ≤ 16 and NAC SSS > 16 (p = 0.021) and SRS ≤ 6 and SRS > 6 (p = 0.003 for AC and p<0.001 for NAC).

Kaplan-Meier curves for all-cause mortality by SSS groups with a cutoff value of 13 for AC and 16 for NAC images were presented in Fig 1A and 1B, which showed significant differences between the two curves for both AC and NAC (p = 0.011 for AC and p = 0.021for NAC). The separation between the two curves began at about two years after MPI. Kaplan-Meier curves for all-cause mortality by SRS groups with a cutoff value of 6 for both AC and NAC images were presented in Fig 1C and 1D, which showed significant differences between the two curves for both AC and NAC (p = 0.003 for AC and p<0.001 for NAC). The separation between the two curves began at about 3 months after MPI and became more obvious at about two years after MPI.

Kaplan-Meier curves for all-cause mortality by SRS 0–3, 4–8, 9–13 and > 13 were presented in S1 Fig which showed significant differences between the curves for both AC and NAC (log-rank test, p = 0.001 and p = 0.005; Wilcoxon test, p<0.001 and p = 0.005 for AC and NAC, respectively). Kaplan-Meier curves for all-cause mortality by SSS 0–3, 4–8, 9–13 and > 13 were also done. Trends of decreased survival with increasing severity of SSS were noted in both AC and NAC images. However, the differences did not reach statistical significance for either AC or NAC (log-rank test, p = 0.07 and 0.09, respectively).

**Fig 2:**
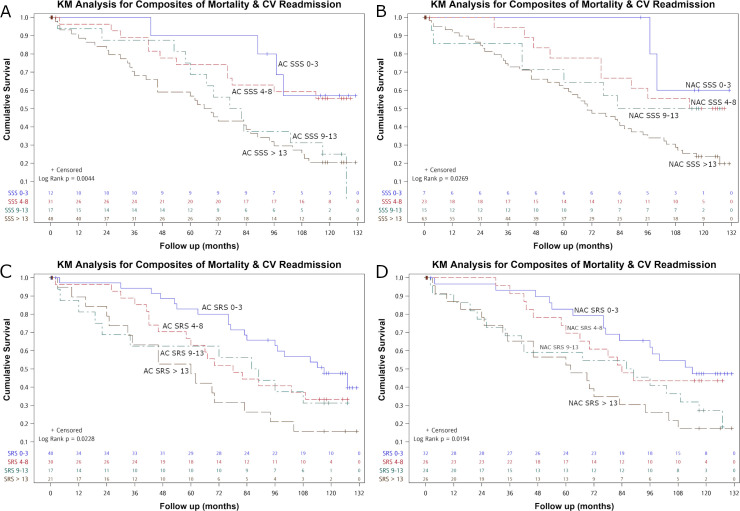
Kaplan-Meier (KM) analysis regarding the composite of all-cause mortality and cardiovascular-related re-admission with SSS 0–3, 4–8, 9–13 and >13 in AC (A), NAC (B), SRS 0–3, 4–8, 9–13 and >13 in AC (C) and NAC (D) images. Significant differences between survivor functions were noted between the SSS four groups (p = 0.004 for AC, 0.027 for NAC) and SRS four groups (p = 0.023 for AC, 0.019 for NAC).

Kaplan-Meier analysis of the composite of all-cause mortality and CV-related re-admission showed significant differences in AC SSS, NAC SSS, AC SRS and NAC SRS, stratified by 0–3, 4–8.9–13, >13, respectively (log rank test, p = 0.004, 0.027, 0.023, 0.019; Wilcoxon test, p = 0.008 and 0.031, 0.009, 0.014, respectively) (Fig 2).

For only CV-related re-admission, although similar trends were noted as with all-cause mortality for SSS, crossover of survival curves became more complicated. As for SRS, the dispersion between the curves further weakened. For HF-related admission, the severity of SSS and SRS could not predict HF admission in either AC or NAC images.

### Cox analysis

The two survival curves for all-cause mortality by SSS of 0–13 versus >13 for AC, SSS 0–16 versus >16 for NAC and SRS 0–6 versus >6 for AC and NAC all showed no crossover, therefore, a Cox proportional hazards model was applied. In the univariable model (Table 3), age, BMI, FRS, AC SSS, NAC SSS, AC SRS, NAC SRS, AC SSS by groups, NAC SSS by groups (0–3, 4–8, 9–13, > 13), AC SRS by groups, NAC SRS by groups (0–3, 4–8, 9–13, > 13), and LVEF were all statistically significant associated with all-cause mortality. However, there were no significant differences in sex, known CAD, number of risk factors, AC SDS, NAC SDS, AC SDS by groups or NAC SDS by groups (0–1, 2–4, 5–8, > 8). For the composite of all-cause mortality plus CV-related re-admission, age, FRS, AC SSS, NAC SSS, AC SRS, NAC SRS, AC SDS, NAC SDS, AC SSS by groups, NAC SSS by groups, AC SRS by groups, NAC SRS by groups and AC SDS by groups all yielded statistically significant results. For CV-related re-admission only, AC SSS, NAC SSS, AC SDS, NAC SDS, and AC SSS by groups yielded statistically significant results and AC SDS by groups yielded borderline significance (p = 0.054). For HF-related admission, LVEF was the only statistically significant protective factor.

**Table 3:** Univariable analysis for all-cause mortality, composite of mortality and cardiovascular (CV)-related re-admission, CV-related re-admission and heart failure (HF) admission.

	Overall Mortality		Composite of Mortality & CV Re-admission		CV Re-admission		HF Admission	
	HR	P value	HR	P value	HR	P value	HR	P value
Gender (male)	0.59	0.17	0.70	0.17	0.71	0.30	0.28	0.17
Age	1.06	0.003	1.04	0.002	1.03	0.12	1.05	0.25
BMI	0.85	0.004	0.97	0.43	1.05	0.24	1.08	0.53
Known CAD	1.79	0.14	1.28	0.32	1.23	0.51	0.86	0.87
Num of Risk Factor	1.31	0.13	1.09	0.45	1.01	0.96	0.76	0.52
FRS (%)	1.06	0.041	1.04	0.032	1.03	0.14	1.08	0.25
AC SSS	1.05	0.013	1.06	<0.001	1.04	0.011	1.04	0.40
NAC SSS	1.06	0.004	1.06	<0.001	1.04	0.013	1.05	0.34
AC SRS	1.07	0.001	1.06	<0.001	1.03	0.28	1.00	0.98
NAC SRS	1.08	<0.001	1.05	<0.001	1.02	0.32	1.00	0.99
AC SDS	1.01	0.80	1.10	<0.001	1.12	<0.001	1.12	0.14
NAC SDS	0.99	0.85	1.08	0.003	1.11	0.001	1.13	0.09
AC SSS group	1.74	0.013	1.05	<0.001	1.37	0.040	1.74	0.27
NAC SSS group	1.92	0.024	1.53	0.005	1.31	0.11	1.65	0.39
AC SRS group	1.79	<0.001	1.38	0.003	1.10	0.51	0.81	0.64
NAC SRS group	1.83	0.001	1.40	0.003	1.15	0.31	1.15	0.72
AC SDS group	1.06	0.75	1.28	0.0495	1.35	0.054	1.70	0.28
NAC SDS group	0.93	0.71	1.22	0.13	1.38	0.07	1.76	0.33
LVEF	0.96	0.002	0.99	0.25	1.01	0.41	0.91	0.003

* SSS group: classifying the severity of MPI by SSS 0–3, 4–8, 8–13, > 13; SRS group: classifying the severity of MPI by SRS 0–3, 4–8, 8–13, > 13; SDS group: classifying the severity of MPI by SDS 0–1, 2–4, 5–8, >8.

** AC: attenuation correction; BMI: body mass index; CAD: coronary artery disease; FRS: Framingham risk score; LVEF: left ventricular ejection fraction; NAC: non-attenuation correction; SSS: summed stress score.

The results of multivariable analysis for SSS were presented in Table 4. After adjusting for sex, age and FRS, BMI was shown to be an independent predictor and LVEF was shown to be a borderline predictor for all-cause mortality in both AC and NAC sets. Only NAC SSS was significantly associated with all-cause mortality (HR: 1.05, p = 0.047), whereas AC SSS was not (p = 0.15). AC SSS and NAC SSS were the only significant predictors for the composite of all-cause mortality plus CV-related re-admission (HR: 1.05, p = 0.001 and < 0.001 for AC and NAC, respectively). For the prediction of CV-related re-admission, SSS showed a statistically significant result (HR: 1.04, p = 0.030 and 0.024 for AC and NAC, respectively) in both AC and NAC sets. For the prediction of HF-related admission, LVEF was the only significant protective independent factor.

**Table 4:** Multivariable analysis for all-cause mortality, composite of mortality and cardiovascular (CV)-related re-admission, CV-related re-admission and heart failure (HF) admission with SSS.

	Overall Mortality		Composite of Mortality & CV Re-admission		CV Re-admission		HF Admission	
	HR	P value	HR	P value	HR	P value	HR	P value
**Clinical data + Attenuation correction**								
**Gender**	0.55	0.17	0.80	0.45	0.76	0.44	0.29	0.22
**Age**	1.02	0.46	1.02	0.23	1.01	0.67	1.04	0.56
**BMI**	0.87	0.028	NA	NA	NA	NA	NA	NA
**FRS(%)**	1.04	0.35	1.01	0.75	NA	NA	NA	NA
**LVEF**	0.97	0.044	NA	NA	NA	NA	0.90	0.009
**AC SSS**	1.04	0.15	1.05	0.001	1.04	0.030	1.03	0.58
**Clinical data + Non-attenuation correction**								
**Gender**	0.53	0.14	0.74	0.32	0.71	0.35	0.24	0.16
**Age**	1.02	0.43	1.02	0.21	1.01	0.63	1.05	0.51
**BMI**	0.88	0.038	NA	NA	NA	NA	NA	NA
**FRS(%)**	1.03	0.37	1.00	0.83	NA	NA	NA	NA
**LVEF**	0.97	0.056	NA	NA	NA	NA	0.90	0.015
**NAC SSS**	1.05	0.047	1.05	<0.001	1.04	0.024	1.06	0.33
**Clinical data + Attenuation correction**								
**Gender**	0.59	0.21	0.88	0.66	0.80	0.53	0.26	0.22
**Age**	1.03	0.39	1.03	0.10	1.01	0.50	1.03	0.69
**BMI**	0.88	0.040	NA	NA	NA	NA	NA	NA
**FRS(%)**	1.03	0.48	1.00	0.87	NA	NA	NA	NA
**LVEF**	0.97	0.055	NA	NA	NA	NA	0.91	0.009
**AC SSS group**	1.30	0.31	1.41	0.011	1.32	0.08	1.43	0.57
**Clinical data + Non-attenuation correction**								
**Gender**	0.54	0.15	0.81	0.48	0.76	0.45	0.21	0.16
**Age**	1.03	0.35	1.03	0.053	1.02	0.33	1.03	0.67
**BMI**	0.88	0.044	NA	NA	NA	NA	NA	NA
**FRS(%)**	1.02	0.53	1.00	0.83	NA	NA	NA	NA
**LVEF**	0.97	0.056	NA	NA	NA	NA	0.90	0.010
**NAC SSS group**	1.61	0.14	1.48	0.011	1.31	0.12	1.84	0.41

AC SSS and NAC SSS were analyzed in two separate models due to significant correlation between AC SSS and NAC SSS.

* SSS group: classifying the severity of MPI by SSS 0–3, 4–8, 8–13, > 13.

** AC: attenuation correction; BMI: body mass index; FRS: Framingham risk score; gr: group; LVEF: left ventricular ejection fraction; NAC: non-attenuation correction; SSS: summed stress score.

After classifying the severity of MPI by SSS 0–3, 4–8, 8–13, > 13, AC SSS by groups and NAC SSS by groups were both significantly associated with the composite of all-cause mortality plus CV-related re-admission (HR: 1.41, p = 0.011 for AC SSS by group; HR: 1.48, p = 0.011 for NAC SSS by group). Only BMI showed a significant protective predictive value (HR: 0.88, p = 0.040; HR: 0.85, p = 0.044, for AC and NAC, respectively) for all-cause mortality in both AC and NAC sets. None of the other predictors were significantly associated with all-cause mortality, the composite of all-cause mortality plus CV-related re-admission, or CV-related re-admission. For the prediction of HF-related admission, LVEF was the only significant protective independent factor (HR: 0.91, p = 0.009; HR: 0.90, p = 0.010, for AC and NAC, respectively).

The results of multivariable analysis for SRS were presented in Table 5. After adjusting for sex, age, FRS and LVEF, BMI and SRS were shown to be independent predictors for all-cause mortality in both AC and NAC sets (HR of SRS: 1.06, p = 0.041 and 1.07 p = 0.009 for AC and NAC, respectively). AC SRS and NAC SRS were the only significant predictors for the composite of all-cause mortality plus CV-related re-admission (HR: 1.05, p = 0.016 and 0.005 for AC and NAC, respectively). After classifying the severity of MPI by SRS 0–3, 4–8, 8–13, > 13, NAC SRS by groups was the only significant predictor for the composite of all-cause mortality plus CV-related re-admission (HR: 1.23, p = 0.035). For the prediction of CV-related re-admission and HF-related admission, SRS showed no statistically significance.

**Table 5:** Multivariable analysis for all-cause mortality, composite of mortality and cardiovascular (CV)-related re-admission, CV-related re-admission and heart failure (HF) admission with SRS.

	Overall Mortality		Composite of Mortality & CV Re-admission		CV Re-admission		HF Admission	
	HR	P value	HR	P value	HR	P value	HR	P value
**Clinical data + Attenuation correction**								
**Gender**	0.59	0.22	0.81	0.47	0.82	0.57	0.42	0.41
**Age**	1.03	0.39	1.03	0.12	1.02	0.31	1.05	0.51
**BMI**	0.86	0.027	NA	NA	NA	NA	NA	NA
**FRS(%)**	1.04	0.33	1.01	0.66	NA	NA	NA	NA
**LVEF**	0.98	0.15	NA	NA	NA	NA	0.90	0.002
**AC SRS**	1.06	0.041	1.05	0.016	1.02	0.48	0.89	0.30
**Clinical data + Non-attenuation correction**								
**Gender**	0.54	0.15	0.75	0.33	0.80	0.54	0.45	0.45
**Age**	1.03	0.38	1.03	0.13	1.02	0.31	1.04	0.53
**BMI**	0.86	0.029	NA	NA	NA	NA	NA	NA
**FRS(%)**	1.04	0.29	1.01	0.70	NA	NA	NA	NA
**LVEF**	0.98	0.34	NA	NA	NA	NA	0.90	0.002
**NAC SRS**	1.07	0.009	1.05	0.005	1.02	0.46	0.92	0.33
**Clinical data + Attenuation correction**								
**Gender**	0.64	0.29	0.88	0.65	0.85	0.63	0.44	0.41
**Age**	1.02	0.41	1.03	0.10	1.02	0.24	1.07	0.38
**BMI**	0.87	0.030	NA	NA	NA	NA	NA	NA
**FRS(%)**	1.03	0.42	1.01	0.62	NA	NA	NA	NA
**LVEF**	0.98	0.17	NA	NA	NA	NA	0.90	0.002
**AC SRS group**	1.45	0.06	1.24	0.06	1.03	0.84	0.46	0.13
**Clinical data + Non-attenuation correction**								
**Gender**	0.60	0.23	0.83	0.51	0.81	0.56	0.47	0.50
**Age**	1.03	0.36	1.03	0.09	1.02	0.32	1.05	0.48
**BMI**	0.87	0.035	NA	NA	NA	NA	NA	NA
**FRS(%)**	1.02	0.52	1.01	0.77	NA	NA	NA	NA
**LVEF**	0.98	0.24	NA	NA	NA	NA	0.90	0.003
**NAC SRS group**	1.51	0.06	1.23	0.035	1.11	0.50	0.69	0.41

AC SRS and NAC SRS were analyzed in two separate models due to significant correlation between AC SRS and NAC SRS.

* SRS group: Classifying the severity of MPI by SRS 0–3, 4–8, 8–13, > 13.

** AC: attenuation correction; BMI: body mass index; FRS: Framingham risk score; gr: group; LVEF: left ventricular ejection fraction; NAC: non-attenuation correction; SRS: summed rest score.

The results of multivariable analysis for SDS were presented in S2 Table. SRS were shown to be the only independent predictor for composite of all-cause mortality plus CV-related re-admission (HR: 1.08, p = 0.006; HR: 1.07, p = 0.012, for AC and NAC, respectively)) and CV-related re-admission (HR: 1.11, p = 0.002; HR: 1.10, p = 0.002, for AC and NAC, respectively) in both AC and NAC sets. However, after classifying the severity of SDS 0–1, 2–4, 5–8, > 8, the significance for predicting composite of all-cause mortality plus CV-related re-admission and CV-related re-admission disappeared.

### Subgroup analysis

Although there was no significant difference between the two Kaplan-Meier survival curves (SSS ≤ 13 and SSS > 13 in AC; SSS ≤ 16 and SSS > 16 in NAC) for all-cause mortality in the obesity (BMI ≥ 27 kg/m^2^) subgroup, there was wider separation between the two curves (S2 Fig) in AC image. However, in the non-obesity subgroup, significant differences between the two survival curves for all-cause mortality and composite of mortality plus CV-related re-admission were noted in both AC and NAC MPI (p = 0.046 and 0.039 for all-cause mortality in AC and NAC, respectively). This implicated that AC and NAC MPI both provided good risk stratification of all-cause mortality in non-obese patients, and AC MPI possibly provided better risk stratification of all-cause mortality in obese patients.

In the discordant subgroup, no patients had an AC SSS above 13. The two Kaplan-Meier survival curves for all-cause mortality and composite of mortality and CV readmission in NAC MPI showed close and crossover pattern. In the concordant subgroup, Kaplan-Meier analysis for all-cause mortality and composite of mortality and CV readmission both showed significant differences between the two curves in AC (p = 0.013 in all-cause mortality; p = 0.011 in composite of mortality and CV readmission). Kaplan-Meier analysis showed borderline difference between the two curves in NAC (p = 0.055) for all-cause mortality and significant difference (p = 0.015) for composite of mortality and CV readmission (S3 Fig). Concordant findings of AC and NAC MPI showed better risk stratification of both all-cause mortality and composite of mortality and CV readmission.

## Discussion

This study showed that with a proper cutoff value of SSS or SRS (SSS 13 for AC, SSS 16 for NAC, SRS 6 for both AC and NAC), there were significant differences in survival in both AC and NAC images. SSS and SRS showed similar independent predictive values in predicting all-cause mortality and composite of all-cause mortality plus CV-related re-admission, in both AC and NAC images. With a trend of SRS better for all-cause mortality and SSS better for composite of all-cause mortality plus CV-related re-admission. In addition, raw SDS data was a good predictor of composite of all-cause mortality plus CV-related re-admission and CV-related re-admission.

SDS demonstrates the ischemic extent of the myocardium, and it has been used for diagnosing CAD. SSS demonstrates the combination of ischemia under stress and scarred myocardium [33]. SRS mainly represents myocardial scar burden. All scores, especially SSS and SDS may change after treatment. In our study, SSS and SRS showed similar predictive values. This may be due to aggressive coronary intervention and optimal medical therapy, such as anti-platelet and statin, in our institute which can modify ischemia. SDS predicted CV-related re-admission, which was also reasonable. In this study, the raw SSS and SRS data mostly showed significant predictive value, however the significance disappeared after classifying by severity using conventional criteria.

Few studies have addressed the prognostic value of AC MPI [11–16]. A recent study by Savvopoulos et al. enrolled 637 patients who underwent CT-based AC Tl-201 MPI and follow-up for 42.3 ± 12.8 months. They concluded that AC was less effective than NAC for risk stratification. In their study, SSS was calculated based on 20 segments. The SSS cutoff values based on the event rate distribution across different SSS were 0–4, 5–13 and >13 for NAC, and 0–2, 3–9 and > 9 for AC. In the multivariate model, abnormal AC yielded no significance for all-cause mortality or the composites of death/non-fatal myocardial infarction and death/myocardial infarction/late revascularization, whereas abnormal NAC was shown to be independent from other covariates for the composite endpoints [15]. The clinical setting of Savvopoulos’ study was very similar to ours. Both used Tl-201 as the imaging agent instead of the widely used technetium-99m (Tc-99m) agents, and both used CT-based AC rather than AC from a line source. Both studies analyzed outcomes from longer follow-up periods instead of 2 years as in most follow-up studies. But, our study had a longer follow-up time of 7.72 ± 3.72 years, comparing with.3.5 years in Savvopoulos’ study. Our study emphasized the similar predictive value effect of SSS and SRS and similar effect of CT-based AC and NAC MPI. The risk stratification power may last longer than previously acknowledged, and interpretation of AC and NAC images combined together can improve diagnostic accuracy [5]. The cutoff values in our study were higher than Savvopoulos’ study, both in original SSS (13 versus 9 for AC and 16 versus 13 for NAC) and percent of diseased myocardium (13/68 versus 9/80 for AC and 16/68 versus 13/80). This may be due to our patient had more severe CAD by inclusion criteria.

A poster only study by Campisi et al. presented a study with 174 patients who underwent Tc-99m sestamibi CT-based AC MPI. Although the final study has not been published as yet, it suggested a similar result that AC MPI did not significantly differ from those by prone imaging [16].

Ardestani et al. and Baghdasarian et al. both reported that AC provided effective risk stratification for future cardiac events in Tc-99m MPI. They both reported that AC-SSS 1–8 and >8 were independent predictors of cardiac events in multivariable analysis (AC-SSS > 8 HR: 7.85, 2.1; p<0.001, 0.0001, respectively) [11, 14]. However, neither Ardestani nor Baghdasarian performed or reported the results of multivariable analysis of NAC data. In our study, both AC and NAC were independent predictors for the composite of all-cause mortality plus CV-related re-admission with similar significance.

Other previous studies have also reported that AC can improve risk stratification for cardiac events. These studies analyzed event rates of NAC and AC MPI in the same SSS severity group or the reclassification rates after AC. The distribution of SSS was usually lower after AC due to a relatively increased uptake after correction, and this may have led to the increase in event rate in the same SSS severity group. A clearer separation between low risk and moderately to severely abnormal findings after AC still confirmed the usefulness of AC. However, comparisons between proportions did not show the independent value of AC or NAC MPI [11–14]. Another study by Zadro et al. explored the prognostic impact of extracardiac findings by CT done for CT-based AC, and reported that patients with major extracardiac findings had poor mid-term outcomes, regardless of ischemic or non-ischemic results of MPI [17].

We tried to find specific patients in whom AC is useful by subgroup analysis. However, only few significant differences were noted in the smaller subgroup analyses, which could be secondary to small sample size and few events. The implication of NAC MPI provided better risk stratification of all-cause mortality in non-obese patients, and AC MPI possibly provided better risk stratification of all-cause mortality in obese patients guide us the future direction of using AC in MPI for outcome prediction. Discordant findings between AC and NAC images implicated that neither AC nor NAC MPI were a good tool for risk stratification.

This study also showed that AC significantly improved diagnostic performance primarily by increasing the specificity. More details in our prior study which subgroup analysis was presented and showed that AC was most helpful in obese subjects, men, and especially inferior wall [5]. Matsuo et al. also reported the value of AC in correcting inferior artifacts in normal subjects and decreased of apical tracer counts was noted after AC [7]. Interpretation of AC and NAC images combined together can lead to more accurate results. We recommended that AC should be applied to Tl-201 MPI as routine clinical practice.

The current study was conducted in an agricultural county with a severe urban-rural gap, and serious population migration, aging problems and inconvenient transportation. Our hospital is the only tertiary referral CV center in this county. This contributed to the low lost to follow-up rate, and only five patients (4.6%) were lost to follow-up during the 10-year longitudinal study follow-up period, who were then contacted via telephone calls. Still, the CV mortality rate was comparable to the whole country. Therefore, the statistical inference of this study can represent the whole country to some degree. All our patients received Tl-201MPI. Although Tl-201 is rarely used in Europe and the US as the radiotracer for MPI currently, it is still commonly used in Taiwan and some Asian countries [34, 35].

Another characteristic of this study was that only patients who received CAG within 90 days in this hospital were recruited, which implied severe or more symptomatic CAD. This was reflected in the outcomes of this cohort, and significantly higher event rates were noted compared with previous studies. The predictive value of MPI is particularly important in these patients, since understanding the prognosis would influence the decision making of cardiologists and health cognition of the patients.

Although this is a retrospective cohort study, all clinical information was recorded using a prospective recording medical report system at the index MPI study, which contained detailed clinical information. In addition, the longer follow-up period decreased the effect of short-term incidental events. Thus, we believe that the long-term prognostic information of CT-based AC on Tl-201 MPI is valuable and convincing.

### Limitations

There were several limitations to this study. First, this was an all-comer, cohort study, but only patients who received CAG within 90 days in this hospital were followed and analyzed. Selection and referral bias might exist and limit the generalization of the results. Patients who received only medical therapy or follow-up tests including CT or invasive coronary angiography > 90 days were not included for analyses, mostly due to asymptomatic or stable symptoms after initial treatments [36]. It suggested that more symptomatic patients were enrolled in this study. Further analyses of the entire cohort would be performed to evaluate the actual prognostic value of CTAC. Second, we evaluated the prognostic value of pre-procedural SSS, SRS and SDS, but, it may change after treatment, especially SSS and SDS. Third, some LVEF data were missing (n = 5). LVEF was a significant variable in univariable analysis and borderline significance in multivariable analysis. Fourth, non-perfusion scintigraphic data or left ventricle volumetric information which has been proven to provide incremental prognostic value was not included in survival model analysis [37–39]. Fifth, our scoring method was not compared to distribution of normal database because there was no Asian databank. Sixth, the SSS, SRS and SDS evaluated by AC MPI might be smaller than non-AC MPI, because AC usually lowered the grading score when attenuation existed. This may cause the statistical analysis result toward the null, causing relative smaller HRs when evaluated by AC MPI. Seventh, small sample size limited the statistical significance, especially in subgroup analysis, and generalization of our conclusion. Finally, some follow-up was done by telephone interview. The number of hospitalizations, detailed causes of hospitalizations and death outside the hospital may not have been precisely recorded using scripted telephone interviews. For example, the rate of HF admission (5/108) was lower than expected, which may have underestimated the predictive value of MPI for HF admission. However, we hoped to emphasize the predictive power of predicting clear hard events, rather than suspected cardiac events.

## Conclusions

Even though AC MPI is traditionally considered to be a better predictor of all-cause mortality than NAC, both CT-based AC and NAC Tl-201 MPI showed similar values. SRS with or without AC were significant predictors for all-cause mortality. SSS and SRS with or without AC were the only significant predictors for the composite of all-cause mortality and CV events in this study.

## Supporting information

S1 Fig Kaplan-Meier (KM) analysis for all-cause mortality by SRS 0–3, 4–8, 9–13 and > 13 in AC and NAC images. (PDF)

S2 Fig Kaplan-Meier (KM) analysis for all cause mortality and cardiovascular (CV)-related re-admission in obesity and non-obesity subgroups. (PDF)

S3 Fig Kaplan-Meier (KM) analysis for all cause mortality and cardiovascular (CV)-related re-admission in subgroups of discordant and concordant findings of AC and NAC. (PDF)

S1 Table Mortality rate by SSS, SRS and SDS groups in AC and NAC images. (PDF)

S2 Table Multivariable analysis for composites of mortality and cardiovascular (CV)-related re-admission and CV-related re-admission with SDS. (PDF)
