# Supplementary material for: Long-term prognostic value of computed tomography-based attenuation correction on thallium-201 myocardial perfusion imaging: A cohort study
Source: PLoS One. 2021 Oct 26;16(10):e0258983. doi: 10.1371/journal.pone.0258983 (PMC8547642; doi:10.1371/journal.pone.0258983)
Supplement: S1 Fig — (PDF) [file pone.0258983.s001.pdf]

S1 Fig. Kaplan-Meier (KM) analysis for all-cause mortality by SRS 0-3, 4-8, 9-13 and > 13 in AC (A) and NAC (B) images, respectively.

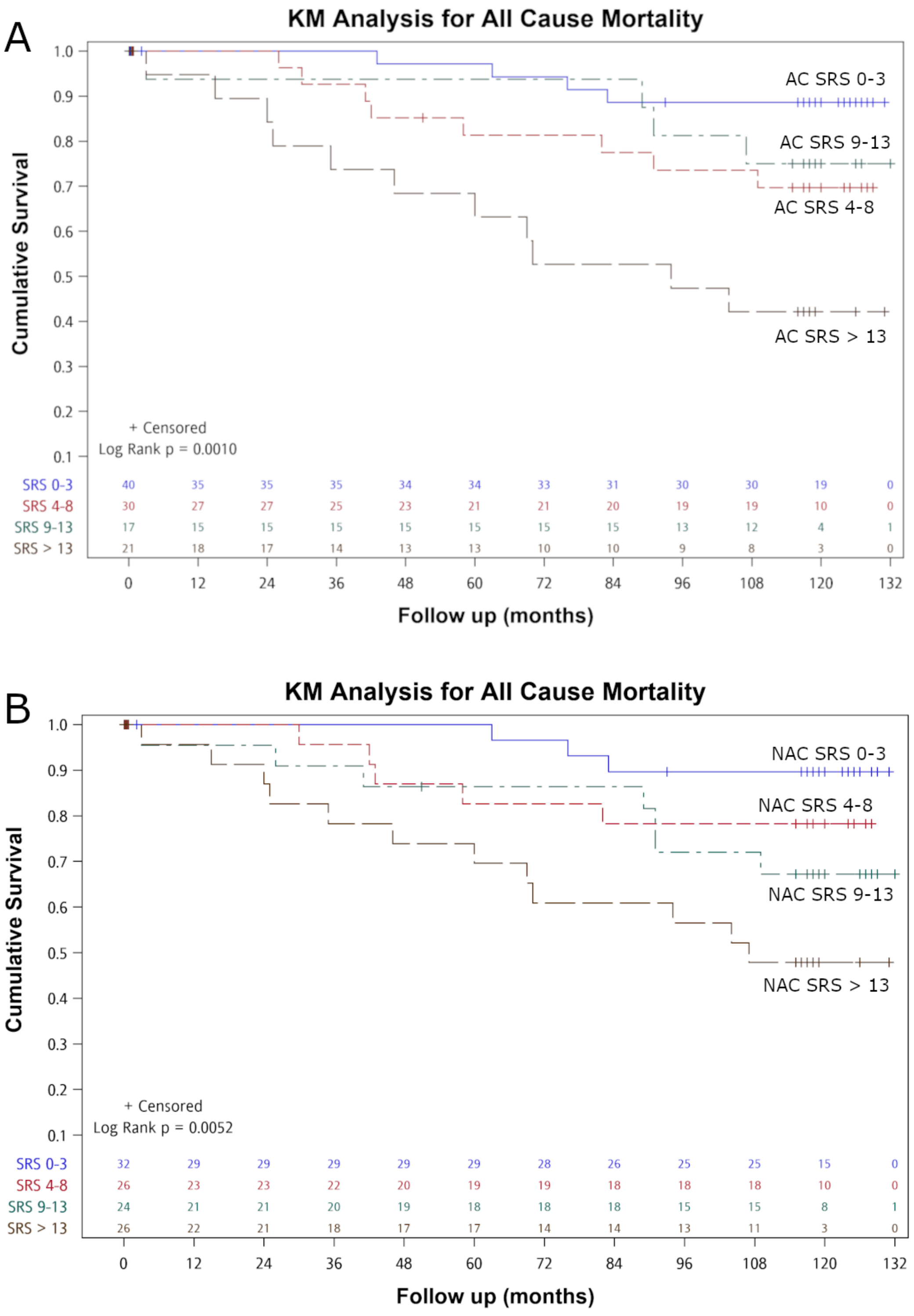

AC: attenuation correction; NAC: non-attenuation correction; SSS: summed stress score
