# Supplementary material for: Long-term prognostic value of computed tomography-based attenuation correction on thallium-201 myocardial perfusion imaging: A cohort study
Source: PLoS One. 2021 Oct 26;16(10):e0258983. doi: 10.1371/journal.pone.0258983 (PMC8547642; doi:10.1371/journal.pone.0258983)
Supplement: S3 Fig — (PDF) [file pone.0258983.s003.pdf]

S3 Fig. Kaplan-Meier (KM) analysis for all cause mortality and composites of mortality and cardiovascular (CV)-related re-admission in subgroups of discordant and concordant findings of AC and NAC.

- Discordant findings of AC and NAC

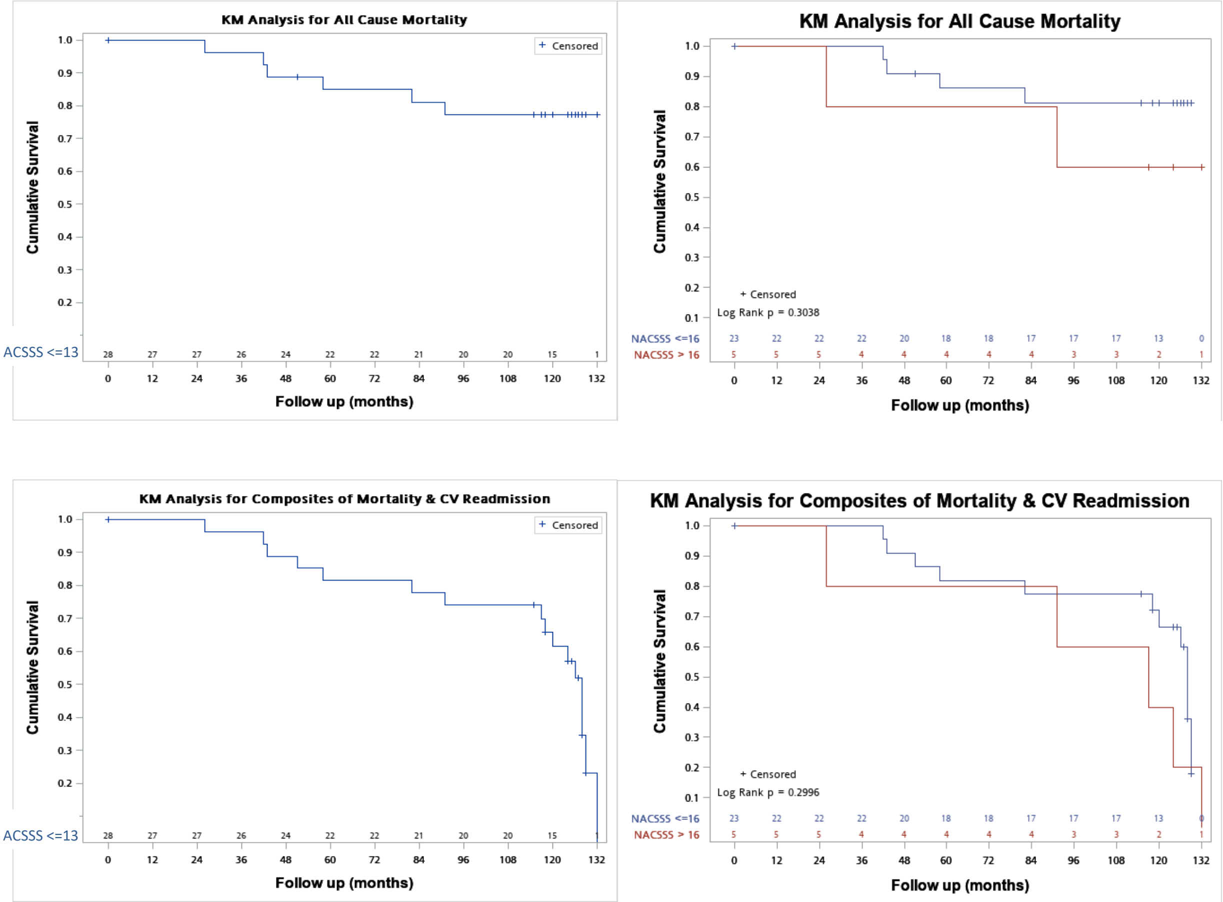

- Concordant findings of AC and NAC

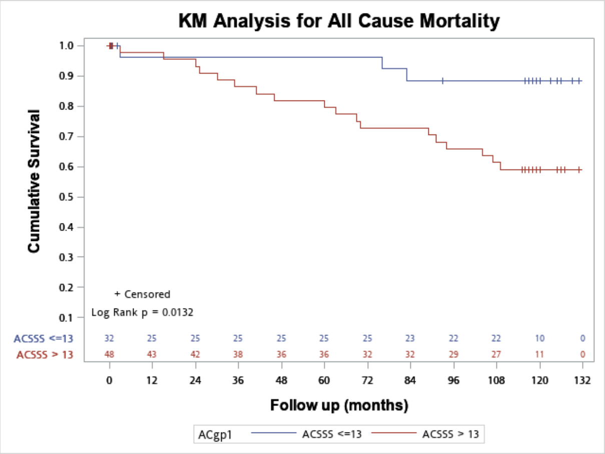

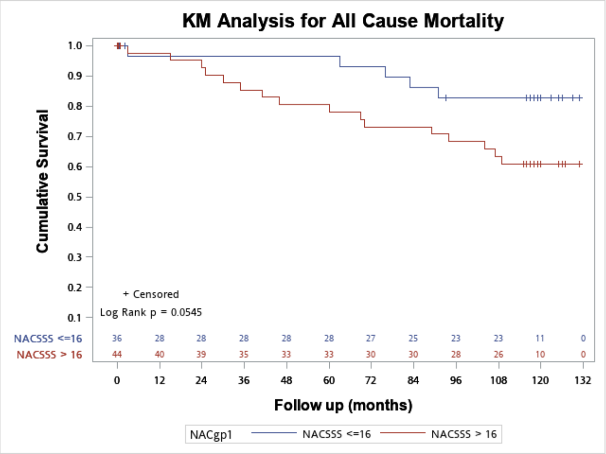

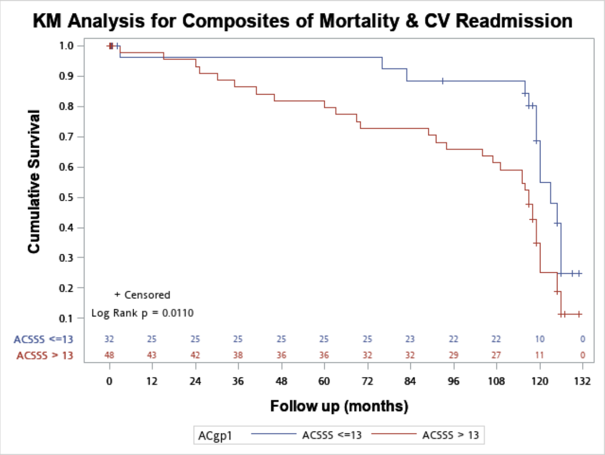

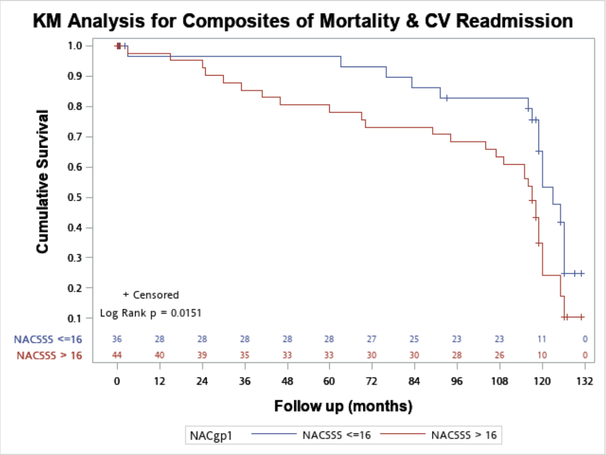

AC: attenuation correction; NAC: non-attenuation correction; SSS: summed stress score
