# Supplementary material for: Long-term prognostic value of computed tomography-based attenuation correction on thallium-201 myocardial perfusion imaging: A cohort study
Source: PLoS One. 2021 Oct 26;16(10):e0258983. doi: 10.1371/journal.pone.0258983 (PMC8547642; doi:10.1371/journal.pone.0258983)
Supplement: S1 Table — (PDF) [file pone.0258983.s004.pdf]

S1 Table. Mortality rate by SSS, SRS and SDS groups in AC and NAC images

SCORE GROUPS		N	PERSON-TIME	MORTALITY	MORTALITY RATE (%) (N/PERSON-TIME)
AC SSS	0-3	12	1120.37	1	0.09%
	4-8	31	2967.34	4	0.13%
	9-13	17	1711.47	4	0.23%
	>13	48	4210.7	18	0.43%
NAC SSS	0-3	7	697.30	0	0.00%
	4-8	23	2044.41	3	0.15%
	9-13	15	1416.47	2	0.14%
	>13	63	5851.7	22	0.38%
AC SRS	0-3	40	4035.11	4	0.10%
	4-8	30	2720.47	8	0.29%
	9-13	17	1736.40	4	0.23%
	>13	21	1517.90	11	0.72%
NAC SRS	0-3	32	3378.77	3	0.09%
	4-8	26	2438.94	5	0.21%
	9-13	24	2215.87	7	0.32%
	>13	26	1976.30	12	0.61%
AC SDS	0-1	21	1591.80	5	0.31%
	2-4	29	2648.14	5	0.19%
	5-8	29	2884.54	8	0.28%
	>8	29	2885.40	9	0.31%
NAC SDS	0-1	15	932.80	4	0.43%
	2-4	23	1909.41	5	0.26%
	5-8	34	3469.60	7	0.20%
	>8	36	3698.07	11	0.30%

AC: attenuation correction; NAC: non-attenuation correction; SDS: summed difference score; SRS: summed rest score; SSS: summed stress score
