# Supplementary material for: Long-term prognostic value of computed tomography-based attenuation correction on thallium-201 myocardial perfusion imaging: A cohort study
Source: PLoS One. 2021 Oct 26;16(10):e0258983. doi: 10.1371/journal.pone.0258983 (PMC8547642; doi:10.1371/journal.pone.0258983)
Supplement: S2 Table — (PDF) [file pone.0258983.s005.pdf]

S2 Table. Multivariable analysis for composites of mortality and cardiovascular (CV)-related re-admission and CV-related re-admission with SDS. AC SDS and NAC SDS were analyzed in two separate models due to significant correlation between AC SDS and NAC SDS.

	**Composite of Mortality & CV Re-admission**		**CV Re-admission**	
	HR	P value	HR	P value
**Clinical data + Attenuation correction**				
**Gender**	0.90	0.71	0.84	0.63
**Age**	1.03	0.11	1.01	0.53
**BMI**	NA	NA	NA	NA
**FRS(%)**	1.01	0.54	NA	NA
**LVEF**	NA	NA	NA	NA
**AC SDS**	1.08	0.006	1.11	0.002
**Clinical data + Non-attenuation correction**				
**Gender**	0.74	0.91	0.86	0.68
**Age**	1.03	0.06	1.02	0.31
**BMI**	NA	NA	NA	NA
**FRS(%)**	1.01	0.52	NA	NA
**LVEF**	NA	NA	NA	NA
**NAC SDS**	1.07	0.012	1.10	0.002
**Clinical data + Attenuation correction**				
**Gender**	0.88	0.66	0.84	0.63
**Age**	1.03	0.07	1.02	0.35
**BMI**	NA	NA	NA	NA
**FRS(%)**	1.02	0.42	NA	NA
**LVEF**	NA	NA	NA	NA
**AC SDS group**	1.17	0.20	1.30	0.10
**Clinical data + Non-attenuation correction**				
**Gender**	0.85	0.58	0.79	0.51
**Age**	1.03	0.054	1.02	0.27
**BMI**	NA	NA	NA	NA
**FRS(%)**	1.02	0.42	NA	NA
**LVEF**	NA	NA	NA	NA
**NAC SDS group**	1.18	0.19	1.36	0.07

* SDS group: classifying the severity of MPI by SDS 0-1, 2-4, 5-8, >8

** AC: attenuation correction; BMI: body mass index; FRS: Framingham risk score; gr: group; LVEF: left ventricular ejection fraction; NAC: non-attenuation correction; SDS: summed difference score
